# Review of the Basics of Cognitive Error in Emergency Medicine: Still No Easy Answers

**DOI:** 10.5811/westjem.2020.7.47832

**Published:** 2020-11-02

**Authors:** Sarah Hartigan, Michelle Brooks, Sarah Hartley, Rebecca E. Miller, Sally A. Santen, Robin R. Hemphill

**Affiliations:** * Virginia Commonwealth University School of Medicine/VCU Health, Department of Internal Medicine, Richmond, Virginia; † University of Michigan, Department of Internal Medicine, Ann Arbor, Michigan; ‡ Virginia Commonwealth University School of Medicine/VCU Health, Department of Emergency Medicine, Richmond, Virginia

## Abstract

Emergency physicians (EP) make clinical decisions multiple times daily. In some instances, medical errors occur due to flaws in the complex process of clinical reasoning and decision-making. Cognitive error can be difficult to identify and is equally difficult to prevent. To reduce the risk of patient harm resulting from errors in critical thinking, it has been proposed that we train physicians to understand and maintain awareness of their thought process, to identify error-prone clinical situations, to recognize predictable vulnerabilities in thinking, and to employ strategies to avert cognitive errors. The first step to this approach is to gain an understanding of how physicians make decisions and what conditions may predispose to faulty decision-making. We review the dual-process theory, which offers a framework to understand both intuitive and analytical reasoning, and to identify the necessary conditions to support optimal cognitive processing. We also discuss systematic deviations from normative reasoning known as cognitive biases, which were first described in cognitive psychology and have been identified as a contributing factor to errors in medicine. Training physicians in common biases and strategies to mitigate their effect is known as debiasing. A variety of debiasing techniques have been proposed for use by clinicians. We sought to review the current evidence supporting the effectiveness of these strategies in the clinical setting. This discussion of improving clinical reasoning is relevant to medical educators as well as practicing EPs engaged in continuing medical education.

## INTRODUCTION

Medical errors are a significant source of harm to patients and distress to physicians. Despite our desire to provide patients with the highest quality of care, rates of medical error remain high with some sources suggesting that diagnostic errors impact about 1 in 20 US adults.^1^,^2^ Several cognitive debiasing strategies have been proposed for reducing diagnostic error.^3^ Many of these techniques focus on how the individual can gain an awareness of their reasoning processes and train their mind to mitigate error from bias. There is real debate as to whether cognitive debiasing is effective. This article will review the existing evidence for using these strategies in the clinical environment, particularly in the emergency department (ED). We will also review theories of cognition and error as well as the research on methods to help decrease rates of medical error related to faulty reasoning.

### Understanding How We Think

To understand how decision-making can lead to medical error, we must first understand how we make decisions. Our current understanding of higher cognitive processes relies on the “dual process theory,” which is a universal model that originated from cognitive psychology and has been applied to the health professions. The theory distinguishes between two systems of thought. System 1 is rapid and intuitive while system 2 is slower and deliberative. Both cognitive systems are critical to decision-making, and each has unique strengths and weaknesses.^4^,^5^ (Table 1).

In most situations, the unconscious, faster, and reflexive system 1 is our default cognitive pathway. This process makes associations between current events and similar past experiences using heuristics, which are cognitive shortcuts or maxims that save time and effort.^6^ System 1 is especially useful in fast-paced, clinical settings like the ED, where it can ease cognitive load and facilitate efficient throughput while reserving working memory.^7^,^8^ A qualitative study of emergency physicians (EP) supported this observation, by demonstrating that most of their diagnostic hypotheses were generated without conscious effort and either prior to or within the first five minutes of an initial patient evaluation.^9^

By contrast, system 2 is deliberative, measured, and analytical. This system uses our working memory to make decisions that require complex problem-solving and greater cognitive effort.^10^ In practice, a physician is not confined to one type of thinking, but instead may alternate between the systems. Expertise develops from repeated use of system 2 thinking, allowing the development of pattern recognition and a subsequent default to system 1 thinking.

### Understanding How We Make Mistakes

Systems 1 and 2 each have potential drawbacks when applied in the clinical setting. Consider the typical process for an EP assessing a new patient. He or she will gather relevant information through history and physical exam, generate differential diagnoses, and use additional testing to narrow the list of possible diagnoses. If the EP uses system 1 thinking, he or she may reach a working diagnosis efficiently using heuristics based on prior experience. For example, a patient with obesity and poorly-controlled diabetes presenting with left leg pain, warmth, and erythema may fit a known pattern of cellulitis. But, the pattern may be applied inappropriately if the EP is inexperienced, key information is missed, or data is misinterpreted.^11^ For example, in the case above, a careful history that details recent surgery and immobilization plus a medication list that includes oral contraceptives may lead the physician to include deep vein thrombosis on the differential. In a review of closed malpractice claims related to a missed or delayed diagnosis in the ED, cognitive factors such as mistakes in judgment were identified in 96% of cases.^12^

System 1 processing is also more prone to error if the patient presentation is complex, evolving, or uncommon.^13^ Greater experience does allow for increased accuracy of system 1 thinking.^14^–^16^ However, more experienced physicians are also more likely to commit to a diagnosis earlier, predisposing them to premature closure and an increased risk of being overconfident in an incorrect diagnosis. This can make it difficult to recognize the need to engage the slower, more deliberate approach of system 2 processing.^17^–^19^

When using system 1, a physician may unconsciously place a higher weight on personal or patient-specific factors. They may over- or underemphasize the significance of a data point to “fit” or exclude a given diagnosis (eg, the lack of pleuritic chest pain means that the shortness of breath is not due to an acute pulmonary embolism). A small study of EPs found that residents were more likely than experienced attendings to reach a diagnosis quickly by discounting or explaining away data that did not “fit” their initial diagnosis.^19^ Likewise, the physician may be influenced by patient-specific biases such as mental illness, obesity, or personality (eg, chest pain in a patient with a psychiatric history is due to anxiety rather than acute coronary syndrome). Additionally, physicians may anchor on a diagnosis due to availability (recently seeing a similar case) or triage bias (going on the diagnosis suggested in triage note). These may also impact the decision to pursue further evaluation or the selection of treatment options.

Despite system 2 being more methodical and systematic, it is not able to detect or correct all the potential cognitive errors of system 1. Furthermore, system 2 has its own vulnerabilities and limitations.^20^ In this deliberate and analytical process, physicians may override their own sound judgments and defer to a physician with more seniority or external resources to guide their decision-making.^11^ When using this system, physicians often generate a broader list of differential diagnoses and employ probability-based approaches to select next steps. Using such an approach will inevitability result in error in the small number of cases where the disease presentation is rare and therefore less likely than a similar but more common diagnosis.^19^ When using system 2, overconfidence can also lead to error. Previous work has shown that lower performers greatly overestimate their abilities. Additionally, they fail to correct their self-assessment even after exposure to the performance of others, resulting in an inability to detect or correct their own errors. Therefore, the ability to engage in self-reflection and recognize one’s own limitations is crucial within this system. 13,21–24 Further, multitasking and taskswitching can lead to errors.

These thought processes are also susceptible to cognitive biases, which are systematic errors that affect decision-making. Bias is relevant to practitioners in emergency medicine who must account for deviations from ideal cognitive processing to arrive at the accurate diagnosis for their patient. Over 100 different cognitive biases have been identified in the literature with nearly 40 described in medicine.^3^,^21^,^25^ For example, availability bias denotes the interpretation of clinical information in the framework of patients seen recently. If a physician recently missed a subarchnoid hemorrhage, he or she may be more likely to think about that diagnosis in the future, whether or not it is relevant to the future case. Bias can also impact other physicians at the time of transition of care. The initial evaluation started in the ED may need to be transitioned to the inpatient setting for ongoing care. The “framing effect” or description of the presentation and current working diagnosis may lead to cognitive bias in the receiving provider and can increase the risk for medical error in the care of these patients.

### What Can We Do to Reduce Cognitive Error?

Strategies to reduce cognitive error in medicine are a growing area of research. Perhaps the most widely accepted approach is to increase expertise through improvement in clinical knowledge and experience.^26^,^27^ This is the essence of training and continuing medical education, but given ongoing rates of error, additional strategies are required.^28^ Various additional approaches have been proposed to decrease errors, but not all have shown benefit in the clinical setting.

### Cognitive Debiasing

One potential solution is debiasing, which targets situations that predispose to bias and offers techniques to avoid errors in clinical reasoning. According to Croskerry, debiasing involves having “the appropriate knowledge of solutions and strategic rules to substitute for a heuristic response” and the ability to override system 1 processing.^6^ For a physician to successfully apply debiasing tactics, he or she must first be aware of common biases and their impact on cognitive error. Then the physician must detect the bias, decide to intervene, and successfully apply strategies to mitigate risk, all the while not becoming paralyzed in decision-making.^29^ Cognitive debiasing offers context-specific rules that substitute for flawed intuitive reasoning while technological debiasing uses external aids to deliver information and reduce cognitive burden.^30^ An example to prevent premature closure might be to review the differential before admitting a patient, or to look for a second fracture when reviewing a hand radiograph, rather than anchoring on the first noted fracture. However, in a study of EM residents, internal medicine residents, and cardiology fellows, a tool to help identify and address cognitive biases in electrocardiogram interpretation had no overall effect in reducing diagnostic errors.^31^

### Increase Clinical Expertise

Effective system 1, non-analytical reasoning relies on both formal and experiential knowledge. With increasing expertise comes the development of exemplars, pattern recognition, or a complex pattern of clinical features representing a diagnosis. These exemplars are stored in a network of associations and connections that facilitate nonanalytic knowledge.^32^ Retrieval of these past associations from memory is less effective in novices who have not yet obtained sufficient experience. Effective training programs and continuing professional development may contribute to the development of a physician’s expertise. Simulation and feedback offer targeted strategies for improving clinical knowledge and experience.^33^,^34^ The success of these strategies relies on the physician’s dedication to the time-intensive practice of identifying and closing gaps in knowledge.

### Awareness of Cognitive Processes and Error Theory

Another strategy to reduce cognitive error is to develop an understanding of the clinical reasoning process and its inherent flaws. This includes knowledge of the major heuristics and biases and an understanding of how they may lead to cognitive error.^35^ Education in these theories has been shown to increase knowledge about cognitive errors. For example, Reilly found that a longitudinal curriculum in diagnostic error and cognitive bias improved recognition and knowledge of cognitive biases by internal medicine residents.^36^ Authors did not explore whether patient errors were reduced. ED faculty who participated in a workshop about biases and debiasing strategies reported improvement in their self-assessed ability to identify common biases encountered in the ED and apply cognitive debiasing strategies to improve diagnostic reasoning.^37^

### Slow-down Strategies

One general error reduction strategy is to encourage physicians to “slow down and be thorough” to allow time for analytical reasoning. The recommendation is that physicians “slow down” when there is something unexpected (cognitive dissonance) or high risk. It is the recognition that the case requires full attention and focus. Multiple studies of this technique have shown little benefit in improving cognitive performance.^38^ As demonstrated by Norman, encouraging residents to slow down during clinical reasoning increased time spent on the task, but had no effect on diagnostic accuracy.^39^ In a trial of EPs and residents, slow conditions and the absence of interruptions also did not improve diagnostic accuracy.^40^ In a randomized controlled trial of trainees and faculty, use of a slow-down strategy while solving bias-inducing clinical vignettes did not improve diagnostic accuracy.^41^ Thus, while it may seem prudent to slow down when the physician does not know an answer, this strategy has not yet proven to be effective.

### Consider Alternatives

The hindsight bias describes how knowledge of an outcome may influence the perception of what actually occurred.^42^ When the outcome of an event is reported, its perceived likelihood increases. “Consider-the-opposite” is a tactic that has been studied in other fields. Considering *what* other outcomes may have occurred and *how* they may have occurred may neutralize the overconfidence that led to the biased judgment.^43^ Considering alternatives may be used as part of slowing down. Hirt and Markman found that asking people to consider any alternative outcome, not only the opposite, had similar benefits.^44^ Evidence for using this strategy to improve clinical reasoning is limited. One study used a novel presentation format to help medical students express their diagnostic reasoning. Students using this technique to present clinical cases offered broader differential diagnosis and provided more justification for their decisions than those using a typical presentation style.^45^ Further investigation is needed to determine the impact of this strategy on diagnostic accuracy.

### Heuristic-based Strategies

Another approach to mitigating bias is to bring attention to the decision-making process and deliberately choose analytic reasoning in situations where the intuitive approach may lead to error. This debiasing technique is known as a cognitive forcing strategy. This strategy can be designed for generic error-prone situations or tailored to a specific clinical context where clinical biases are frequently seen.^35^ There has been mixed success with this approach in the cognitive laboratory setting. EM residents who experienced a simulation of cognitive error traps followed by didactics on cognitive forcing strategies self-reported increased knowledge about cognitive strategies and heuristic techniques.^46^ Additionally, the use of a mnemonic checklist to facilitate metacognition and cognitive debiasing improved diagnostic decision-making by medical students in case scenarios.^47^

Jenkins performed a randomized trial to improve diagnosis in pediatric bipolar disorder. Mental health professionals trained in cognitive errors and debiasing strategies made fewer diagnostic errors and demonstrated higher diagnostic accuracy in clinical vignettes designed to test for specific cognitive errors.^48^ But Sherbino found that training in the use of cognitive forcing strategies did not reduce diagnostic errors by medical students in computer-based cases.^49^ Smith and Slack designed a workshop that introduced family medicine residents to cognitive error and debiasing techniques. Trained faculty helped learners apply these concepts to patients in clinic visits involving a new diagnosis. The intervention did not increase the residents’ ability to recognize their risk of cognitive bias in the clinical setting.^50^

While there is evidence that physicians can gain knowledge of clinical biases, there is less evidence that they can recognize biases in practice. Recognizing and mitigating biases is a challenge given that they occur during decision-making at the subconscious level.^32^ It is uncertain whether debiasing approaches can be effective at reducing cognitive error in the clinical setting.^34^

### Reflective Practice

Reflective practice, also known as a diagnostic “time out,” is a strategy to promote metacognition. The practice involves re-evaluating experience and considering alternatives to produce insights with the potential to change behavior in future practice.^33^ In one study using this strategy, medical students were asked to review case-based scenarios and offer an initial diagnosis. Next, they were asked to reflect on and revise their initial diagnoses, resulting in minimal incremental benefits to diagnostic accuracy.^51^ Mamede et al had medical students and residents diagnose clinical cases under conditions that promoted unconscious and conscious deliberation. With residents, this strategy led to improved diagnostic accuracy on complex cases. However, medical students demonstrated worse diagnostic accuracy under the same conditions.^52^ It is unclear whether the benefits seen with residents were due to reducing bias, or just allowing additional time for assessment.

In another study by the same author, reflective reasoning counteracted diagnostic error due to the availability bias in internal medicine residents.^53^ Hospitalists who used a guided reflective-practice tool to review patient readmissions changed their discharge planning behaviors and experienced a sustained reduction in 30-day readmissions.^54^ Given that the benefits of reflective practice were demonstrated with residents and physicians, but not students, it is possible that adequate background knowledge is a prerequisite for success of this strategy. Further study is needed to determine whether this strategy can be successful for junior learners, or if it is a more advanced strategy that should be reserved for those with more clinical expertise.

### Second Opinions

One method to address errors is to obtain additional expertise through consultation. While the contribution of others may be helpful it is important to not be over-reliant on an authoritative consult. Obtaining a second opinion had a variable impact on identifying errors in studies involving interpretation of pathology specimens and radiographic images.^25^ In one successful study, Duijm demonstrated that additional independent readings of screening mammograms resulted in a modest increase in breast cancer detection rates.^55^ Other related strategies include consulting and learning from experts and relying on the collective wisdom gained through group decision-making.^33^ For example, in a recent study of EPs, use of systematic cross-checks was associated with a decreased risk of adverse events.^56^

### Checklists, Guidelines and Algorithms

When physicians experience high levels of stress and fatigue, cognitive function can suffer. Checklists are effective tools for reducing error in these environments by reducing reliance on memory, but can also help minimize cognitive errors. Checklists may serve a variety of purposes, including assisting with diagnosis, ensuring standardization, and providing reminders of evidence-based practice. Evidence shows that checklists not only reduce error but also improve outcomes.^57^ For example, Haynes demonstrated that implementation of a surgical safety checklist reduced complications and in-hospital mortality.^58^ In EM, there are mental checklists for intubation, central line insertion, and other domains. Similarly, clinical guidelines and algorithms may support decision-making in situations prone to error.^33^ For example “MUDPILES” as the mnemonic for anion- gap acid-base disorders helps to ensure considering a broad differential.

### Clinical Decision Support

Clinical decision support systems (CDSS) analyze data to provide physicians with recommendations that aid clinical decision-making. For example, CDSS can detect early evidence of clinical deterioration or give alerts about potentially dangerous drug interactions. These systems have been shown to reduce medication errors and improve adherence to best practice.^59^,^60^ However, systematic reviews of these systems suggest that not all CDSS are successful. Features of the most effective CDSS include the following: the system is computer-based; it offers actionable recommendations; it gives support at the time and location of decision- making; and it functions automatically within the physician workflow.^59^

## LIMITATIONS

There are limitations to our understanding of clinical reasoning and cognitive debiasing. Many of the suggested strategies for reducing cognitive error in medicine are drawn from evidence in other fields. The evidence on reducing errors in clinical reasoning is drawn from mostly single-center studies with small sample sizes and lack of randomization. Most studies enrolled medical students or residents, leaving gaps in knowledge regarding effectiveness of these strategies for practicing physicians. Intervention studies mainly involved laboratory settings, raising questions about the potential impact of these techniques in the clinical environment.

## CONCLUSION

Mistakes in diagnosis are a considerable source of error in medicine. The clinical reasoning process includes dual-process theory, which includes both intuitive and analytical reasoning. A broad array of interventions has been proposed to reduce cognitive error in medicine, but evidence regarding the effectiveness of these strategies in the healthcare setting is limited.^61^–^62^ In particular, there is not yet strong evidence to support a reduction in cognitive errors by bringing attention to error-prone clinical situations and offering tools to mitigate bias. Techniques that reduce cognitive burden through technological or other external means offer some promise and warrant further investigation. Strategies to reduce cognitive error are a growing area of research.

## Figures and Tables

**Table 1:** Comparison of the dual-process theory of thought: system 1 (intuition) and system 2 (analytic)^5^,^7^,^8^

Intuition (system 1)	Analytic (system 2)
Familiar situations	Uncertain, unfamiliar, or undifferentiated situations
Relies on prior experience/training	Relies on pursuit of new knowledge/information
Relatively fast	Relatively slow
Efficient, time-sparing	Rigorous, time-consuming
Unconscious, automatic	Deliberate, controlled
Pattern recognition, heuristics, associations	Logical, analytical, rule-based, hypotheticodeductive method
Default system	Activated when needed (eg, high-stakes situations or complex presentations) or when time permits
Requires context, personalized	Decontextualized, depersonalized
Interactional intelligence	Analytic intelligence

## References

[1] Singh H, Meyer AN, Thomas EJ (2014). The frequency of diagnostic errors in outpatient care: estimations from three large observational studies involving US adult populations. BMJ Qual Saf.

[2] Graber ML (2013). The incidence of diagnostic error in medicine. BMJ Quality & Safety.

[3] Croskerry P (2003). The importance of cognitive errors in diagnosis and strategies to minimize them. Acad Med.

[4] Pelaccia T, Tardif J, Triby E (2011). An analysis of clinical reasoning through a recent and comprehensive approach: the dual-process theory. Med Educ Online.

[5] Croskerry P (2009). A universal model of diagnostic reasoning. Acad Med.

[6] Croskerry P, Singhal G, Mamede S (2013). Cognitive debiasing 1: origins of bias and theory of debiasing. BMJ Qual Saf.

[7] Epstein S (1994). Integration of the cognitive and the psychodynamic unconscious. Am Psychol.

[8] Pelaccia T, Messman AM, Kline JA (2020). Misdiagnosis and failure to diagnose in emergency care: causes and empathy as a solution. Patient Educ Couns.

[9] Pelaccia T, Tardif J, Triby E (2014). How and when do expert emergency physicians generate and evaluate diagnostic hypotheses? A qualitative study using head-mounted video cued-recall interviews. Ann Emerg Med.

[10] Kahneman D, Frederick S, Gilovich T, Griffin D, Kahneman D (2002). Representativeness revisited: attribute substitution in intuitive judgment. Heuristics and Biases: The Psychology of Intuitive Judgment.

[11] Bordini BJ, Stephany A, Kliegman R (2017). Overcoming diagnostic errors in medical practice. J Pediatr.

[12] Kachalia A, Gandhi TK, Puopolo AL (2007). Missed and delayed diagnoses in the emergency department: a study of closed malpractice claims from 4 liability insurers. Ann Emerg Med.

[13] Ely JW, Levinson W, Elder NC (1995). Perceived causes of family physicians’ errors. J Fam Pract.

[14] Groves M, Scott I, Alexander H (2002). Assessing clinical reasoning: a method to monitor its development in a PBL curriculum. Med Teach.

[15] Monteiro SD, Sherbino JD, Ilgen JS (2015). Disrupting diagnostic reasoning: Do interruptions, instructions, and experience affect the diagnostic accuracy and response time of residents and emergency physicians?. Acad Med.

[16] ALQahtani DA, Rotgans JI, Mamede S (2016). Does time pressure have a negative effect on diagnostic accuracy?. Acad Med.

[17] Hobu PP, Schmidt HG, Boshuizen HP (1987). Contextual factors in the activation of first diagnostic hypotheses: expert-novice differences. Med Educ.

[18] Friedman CP, Gatti GG, Franz TM (2005). Do physicians know when their diagnoses are correct?. J Gen Intern Med.

[19] Schubert CC, Denmark TK, Crandall B (2013). Characterizing novice-expert differences in macrocognition: an exploratory study of cognitive work in the emergency department. Ann Emerg Med.

[20] Evans JSB, Stanovich KE (2013). Dual-process theories of higher cognition: advancing the debate. Perspect Psychol Sci.

[21] Graber M, Gordon R, Franklin N (2002). Reducing diagnostic errors in medicine. Acad Med.

[22] Kruger J, Dunning D (1999). Unskilled and unaware of it: how difficulties in recognizing one’s own incompetence lead to inflated self-assessments. J Pers Soc Psychol.

[23] Saposnik G, Redelmeier D, Ruff C (2016). Cognitive biases associated with medical decisions: a systematic review. BMC Medical Informatics and Decision Making.

[24] Skaugset LM, Farrell S, Carney M (2016). Can you multitask? Evidence and limitations of task switching and multitasking in emergency medicine. Ann Emerg Med.

[25] Norman GR, Monteiro SD, Sherbino J (2017). The causes of errors in clinical reasoning: cognitive biases, knowledge deficits, and dual process thinking. Acad Med.

[26] Graber ML, Kissam S, Payne VL (2012). Cognitive interventions to reduce diagnostic error: a narrative review. BMJ Qual Saf.

[27] Berk WA, Welch RD, Levy PD (2008). The effect of clinical experience on the error rate of emergency physicians. Ann Emerg Med.

[28] Royce CS, Hayes MM, Schwartzstein RM (2018). Teaching critical thinking: a case for instruction in cognitive biases to reduce diagnostic errors and improve patient safety. Acad Med.

[29] Croskerry P, Singhal G, Mamede S (2013). Cognitive debiasing 2: impediments to and strategies for change. BMJ Qual Saf.

[30] Ludolph R, Schulz PJ (2018). Debiasing health-related judgments and decision making: A systematic review. Med Decis Making.

[31] Sibbald M, Sherbino J, Ilgen JS (2019). Debiasing versus knowledge retrieval checklists to reduce diagnostic error in ECG interpretation. Adv Health Sci Educ Theory Pract.

[32] Monteiro S, Norman G, Sherbino J (2018). The 3 faces of clinical reasoning: epistemological explorations of disparate error reduction strategies. J Eval Clin Pract.

[33] Thammasitboon S, Cutrer WB (2013). Diagnostic decision making and strategies to improve diagnosis. Curr Probl Pediatr Adolesc Health Care.

[34] Scott I (2009). Errors in clinical reasoning: causes and remedial strategies. BMJ.

[35] Croskerry P (2003). Cognitive forcing strategies in clinical decisionmaking. Ann Emerg Med.

[36] Reilly JB, Ogdie AR, Von Feldt JM (2013). Teaching about how doctors think: a longitudinal curriculum in cognitive bias and diagnostic error for residents. BMJ Qual Saf.

[37] Daniel M, Carney M, Khandelwal S (2017). Cognitive debiasing strategies: a faculty development workshop for clinical teachers in emergency medicine. MedEdPORTAL.

[38] Norman GR, Monteiro SD, Sherbino J (2017). The causes of errors in clinical reasoning: cognitive biases, knowledge deficits, and dual process thinking. Acad Med.

[39] Norman G, Sherbino J, Dore K (2014). The etiology of diagnostic errors: a controlled trial of system 1 versus system 2 reasoning. Acad Med.

[40] Monteiro SD, Sherbino JD, Ilgen JS (2015). Disrupting diagnostic reasoning: do interruptions, instructions, and experience affect the diagnostic accuracy and response time of residents and emergency physicians?. Acad Med.

[41] O’Sullivan E, Susie Shofield (2019). A cognitive forcing tool to mitigate cognitive bias: a randomized control trial. BMC Med Educ.

[42] Croskerry P (2003). The importance of cognitive errors in diagnosis and strategies to minimize them. Acad Med.

[43] Slovic P, Fischhoff B (1977). On the psychology of experimental surprises. J Exp Psychol.

[44] Hirt ER, Markman KD (1995). Multiple explanation: a consider-an-alternative strategy for debiasing judgements. J Pers Soc Psychol.

[45] Wolpaw T, Papp KK, Bordage G (2009). Using SNAPPS to facilitate the expression of clinical reasoning and uncertainties: a randomized comparison group trial. Acad Med.

[46] Bond WF, Deitrick LW, Arnold DC (2004). Using simulation to instruct emergency medicine residents in cognitive forcing strategies. Acad Med.

[47] Chew KS, Durning SJ, van Merrienboer JJG (2016). Teaching metagognition in clinical decision-making using a novel mnemonic checklist: an exploratory study. Singapore Med J.

[48] Jenkins MM, Youngstrom EA (2016). A randomized controlled trial of cognitive debiasing improves assessment and treatment selection for pediatric bipolar disorder. J Consult Clin Psychol.

[49] Sherbino J, Kulasegaram K, Howey E (2014). Ineffectiveness of cognitive forcing strategies to reduce biases in diagnostic reasoning: a controlled trial. CJEM.

[50] Smith BW, Slack MB (2015). The effect of cognitive debiasing training among family medicine residents. Diagnosis (Berl).

[51] Monteiro SD, Sherbino J, Patel A (2015). Reflecting on diagnostic errors: taking a second look is not enough. J Gen Intern Med.

[52] Mamede S, Schmidt HG, Rikers RMJP (2010). Conscious thought beats deliberation without attention in diagnostic decision-making: at least when you are an expert. Psych Research.

[53] Mamede S, van Gog T, van der Berge K (2010). Effect of availability bias and reflective reasoning on diagnostic accuracy among internal medicine residents. JAMA.

[54] Kashiwagi DT, Burton MC, Hakim FA (2016). Reflective practice: a tool for readmission reduction. Am J of Med Qual.

[55] Duijm LE, Groenewoud JH, Fracheboud J (2007). Additional double reading of screening mammograms by radiologic technologist: impact on screening performance parameters. J Natl Cancer Inst.

[56] Freund Y, Goulet H, Leblanc J (2018). Effect of systematic physician cross-checking on reducing adverse events in the emergency department: the CHARMED cluster randomized trial. JAMA Intern Med.

[57] Hales BM, Pronovost PJ (2006). The checklist a tool for error management and performance improvement. J of Crit Care.

[58] Haynes AB, Berry WR, Breizat AHS (2009). A surgical safety checklist to reduce morbidity and mortality in a global population. N Engl J Med.

[59] Kawamoto K, Houlihan CA, Balas EA (2005). Improving clinical practice using clinical decision support systems: a systematic review of trials to identify features critical to success. BMJ.

[60] Kaushal R, Shojania KG, Bates DW (2003). Effects of computerized physician order entry and clinical decision support systems on medication safety. Arch Intern Med.

[61] Lambe KA, O’Reilly G, Kelly BD (2016). Dual-process cognitive interventions to enhance diagnostic reasoning: a systematic review. BMJ Qual Saf.

[62] O’Sullivan E, Susie Shofield (2019). A cognitive forcing tool to mitigate cognitive bias- a randomized control trial. BMC Med Educ.

